# Supplementary material for: COZOID: contact zone identifier for visual analysis of protein-protein interactions
Source: BMC Bioinformatics. 2018 Apr 6;19:125. doi: 10.1186/s12859-018-2113-6 (PMC5889581; doi:10.1186/s12859-018-2113-6)
Supplement: Supplementary file 2 — Software build. Executable binary file of the software tool. (ZIP 118,784 kb) [file 12859_2018_2113_MOESM2_ESM.zip › cozoid/java/docs/beansbinding-1.2.1-doc.zip!/org/jdesktop/observablecollections/ObservableList.html]

ObservableList (Beans Binding)


---


|  |  |  |
| --- | --- | --- |
| **Overview** \| **Package** \| **Class** \| **Use** \| **Tree** \| **Deprecated** \| **Index** \| **Help** | |  |
| **PREV CLASS**   **NEXT CLASS** | **FRAMES**    **NO FRAMES**     **All Classes** |  |
| SUMMARY: NESTED \| FIELD \| CONSTR \| METHOD | DETAIL: FIELD \| CONSTR \| METHOD |  |


---


## org.jdesktop.observablecollections Interface ObservableList<E>

**All Superinterfaces:**: java.util.Collection<E>, java.lang.Iterable<E>, java.util.List<E>

---

``` public interface ObservableList<E> extends java.util.List<E> ```

A `List` that notifies listeners of changes.

---

| **Method Summary** | |
| --- | --- |
| `void` | `addObservableListListener(ObservableListListener listener)`             Adds a listener that is notified when the list changes. |
| `void` | `removeObservableListListener(ObservableListListener listener)`             Removes a listener. |
| `boolean` | `supportsElementPropertyChanged()`             Returns `true` if this list sends out notification when the properties of an element change. |

**Methods inherited from interface java.util.List**

`add, add, addAll, addAll, clear, contains, containsAll, equals, get, hashCode, indexOf, isEmpty, iterator, lastIndexOf, listIterator, listIterator, remove, remove, removeAll, retainAll, set, size, subList, toArray, toArray`

**Method Detail**

### addObservableListListener

```
void addObservableListListener(ObservableListListener listener)
```

:   Adds a listener that is notified when the list changes.

    :   **Parameters:**: `listener` - the listener to add

---


### removeObservableListListener

```
void removeObservableListListener(ObservableListListener listener)
```

:   Removes a listener.

    :   **Parameters:**: `listener` - the listener to remove

---


### supportsElementPropertyChanged

```
boolean supportsElementPropertyChanged()
```

:   Returns `true` if this list sends out notification when
    the properties of an element change. This method may be used
    to determine if a listener needs to be installed on each of
    the elements of the list.

    :   **Returns:**: `true` if this list sends out notification when the properties of an element change


---


|  |  |  |
| --- | --- | --- |
| **Overview** \| **Package** \| **Class** \| **Use** \| **Tree** \| **Deprecated** \| **Index** \| **Help** | |  |
| **PREV CLASS**   **NEXT CLASS** | **FRAMES**    **NO FRAMES**     **All Classes** |  |
| SUMMARY: NESTED \| FIELD \| CONSTR \| METHOD | DETAIL: FIELD \| CONSTR \| METHOD |  |


---
